# Evaluation of reader performance during interpretation of breast cancer screening: the *Recall and detection Of breast Cancer in Screening* (ROCS) trial study design

**DOI:** 10.1007/s00330-022-08820-5

**Published:** 2022-04-28

**Authors:** Ioannis Sechopoulos, Craig K. Abbey, Daniëlle van der Waal, Tanya Geertse, Eric Tetteroo, Ruud M. Pijnappel, Mireille J.M. Broeders

**Affiliations:** 1 Department of Medical Imaging, Radboud University Medical Center, P.O. Box 9101 (766), 6500 HB Nijmegen, the Netherlands; 2 Dutch Expert Centre for Screening (LRCB), Nijmegen, the Netherlands; 3 Technical Medicine Center, University of Twente, Enschede, the Netherlands; 4 Department of Psychological and Brain Sciences, University of California Santa Barbara, Santa Barbara, CA USA; 5 Amphia Hospital, Breda, the Netherlands; 6 Department of Radiology, University Medical Center Utrecht, Utrecht University, Utrecht, the Netherlands; 7 Department for Health Evidence, Radboud University Medical Center, Nijmegen, the Netherlands

**Keywords:** Mammography, Cancer screening, Breast cancer, ROC analysis, Task performance and analysis

## Abstract

**Abstract:**

The magnitude of the tradeoff between recall rate (RR) and cancer detection rate (CDR) in breast-cancer screening is not clear, and it is expected to depend on target population and screening program characteristics. Multi-reader multi-case research studies, which may be used to estimate this tradeoff, rely on enriched datasets with artificially high prevalence rates, which may bias the results. Furthermore, readers participating in research studies are subject to “laboratory” effects, which can alter their performance relative to actual practice. The *Recall and detection Of breast Cancer in Screening* (ROCS) trial uses a novel data acquisition system that minimizes these limitations while obtaining an estimate of the RR-CDR curve during actual practice in the Dutch National Breast Cancer Screening Program. ROCS involves collection of at least 40,000 probability-of-malignancy ratings from at least 20 radiologists during interpretation of approximately 2,000 digital mammography screening cases each. With the use of custom-built software on a tablet, and a webcam, this data was obtained in the usual reading environment with minimal workflow disruption and without electronic access to the review workstation software. Comparison of the results to short- and medium-term follow-up allows for estimation of the RR-CDR and receiver operating characteristics curves, respectively. The anticipated result of the study is that performance-based evidence from practice will be available to determine the optimal operating point for breast-cancer screening. In addition, this data will be useful as a benchmark when evaluating the impact of potential new screening technologies, such as digital breast tomosynthesis or artificial intelligence.

**Key Points:**

*• The ROCS trial aims to estimate the recall rate–cancer detection rate curve during actual screening practice in the Dutch National Breast Cancer Screening Program.*

*• The study design is aimed at avoiding the influence of the “laboratory effect” in usual observer performance studies.*

*• The use of a tablet and a webcam allows for the acquisition of probability of malignancy ratings without access to the review workstation software.*

**Supplementary Information:**

The online version contains supplementary material available at 10.1007/s00330-022-08820-5.

## Introduction

Combined with improvements in treatment, mammography-based screening has been credited with an important decrease in breast cancer mortality over the last two decades [1]. Nonetheless, screening mammography itself is not a perfect diagnostic test; it invariably involves a tradeoff between sensitivity and specificity, or equivalently, the unconditional outcomes of recall and detection rates. Ideally, when screening is performed as part of a regional or national program, this tradeoff should be selected to balance the outcomes according to the priorities of the program, the characteristics of the target population, and the procedures used for screening.

In the Netherlands, a publication by Otten et al [2] has been highly influential in establishing guidelines for radiologist performance in screening mammography. This study led the screening program to adopt a target recall rate of 2%, with a predicted cancer detection rate of 4.52 women per 1000 screens. These guidelines remained in place as the program transitioned to digital mammography (DM). Shortly after this transition, in 2011, the recall rate was close to this value (2.14% with 95% CI: 2.12–2.17%), although the detection rate (6.3/1000 with 95% CI: 6.2–6.5) was 40% higher than what was anticipated from the Otten study. This difference may be attributable to the “laboratory” nature of the study, in which a curated set of cases was used to evaluate reader performance with the knowledge that reader decisions did not involve patient management.

Over the years since, the recall rate in the Netherlands has varied, typically with somewhat higher observed values than the recommendation. In 2016, the program recall rate was 2.43%, with a corresponding detection rate to 7.0 cancers per 1000 women screened [3]. In the latest available complete statistics, from 2018, the recall rate has decreased to 2.23% with a corresponding cancer detection rate of 6.7/1000 [3]. These deviations from the expected recall and cancer-detection levels, combined with interest in new approaches to screening, such as artificial intelligence and digital breast tomosynthesis, have motivated further examination of the tradeoff between recall and cancer-detection rates in breast cancer screening. The purpose of the *Recall and detection Of breast Cancer in Screening* (ROCS) trial is to characterize the underlying relationship between these measures in screening practice.

Audit data from screening practice is commonly used for determining program performance. However, audit data produces a single estimate of the recall/detection pair, and therefore has limited value for evaluating the tradeoff between recall and detection rates. To evaluate this tradeoff, a probability of malignancy (PoM) score is needed that can be compared to different thresholds for recall, but these are not obtained as part of screening practice. In essence, this PoM reflects the reader’s internal decision process, before applying a threshold to dichotomize their decision into a recall/no-recall outcome [4–6]. Laboratory studies allow for collection of PoM scores, which are then used to generate receiver operating characteristic (ROC) curves that quantify the tradeoff between sensitivity and specificity. ROC performance can be readily converted into estimates of recall and detection rates with knowledge of disease prevalence. However, these studies are potentially subject to so-called *laboratory effects* that may lead to biased results. Typically, laboratory studies involve the enrichment of the testing set with additional examples of positive cases, which can also lead to prevalence biases in reader responses [7].

The ROCS trial avoids the potential issue of laboratory effects and the difficulty of extrapolating current performance into predictions of performance at different operating points by using a novel system to acquire PoM scores during actual screening practice. The system is designed to minimally interrupt clinical workflow, and to automatically associate the PoM response with the case identifier. In this Special Report, we present the design of the ROCS trial, aimed at determining the performance tradeoffs for digital mammography interpretation in the actual breast cancer screening environment of the Dutch National Breast Cancer Screening Program. At present, the first stage of data acquisition, the accrual of reader case scores during screening is completed, and the results of this stage are presented. The second and final stages of data acquisition, involving the determination of the ground truth for the recalled cases and, then, after the 2-year waiting period, for the non-recalled cases, are ongoing.

## Materials and methods

No external funding was needed or obtained for the performance of this study. The study was waived of the requirement of ethics approval and informed consent by the research ethics committee of the Radboud University Medical Center.

### Overall study design

The primary aim of the ROCS trial is to estimate the trade-off between recall rate (RR) and cancer detection rate (CDR) by estimating the RR-CDR curve for the average radiologist in the Dutch National Breast Cancer Screening Program directly from screening interpretations. Secondary endpoints include estimating the ROC curve for the average breast radiologist during actual screening interpretation and determining if reader performance changes as they progress within one reading session, as previously reported [8].

For this, ROCS involves obtaining a PoM rating from screening radiologists (*n* = 20) for each and every case (*n* = 2,000 per radiologist) they interpret during several screening reading sessions (approximately *n* = 8), with minimal interruption or diversion from their usual reading workflow. The outcomes of the cases included in the ROCS trial will be obtained from short-term (1 year) follow-up of the recalled cases via the screening organization and long-term (2 years) follow-up via the Netherlands Cancer Registry.

### Study cases

The digital mammography cases included in ROCS involved cases acquired and interpreted as part of the Dutch National Breast Cancer Screening Program. In the Netherlands, the screening program invites all women between the ages of 50 and 75 to be screened for breast cancer with digital mammography nominally every 2 years, with this period having some regional variability. All image acquisitions, involving the standard cranio-caudal (CC) and medio-lateral oblique (MLO) views, are performed at dedicated screening centers equipped with the same imaging systems (Selenia, Hologic Inc.). The systems are all under the same quality control protocol, performed by the same group of physicists at the Dutch Expert Centre for Screening (LRCB) for the nationwide program. The technologists that acquire all images are specifically trained and undergo regular refresher education and auditing on screening mammography. Cases are interpreted in batches, at dedicated reading centers, usually within 1 or 2 days of acquisition. All reading is performed by certified screening radiologists, who undergo additional periodic training and evaluation to be a part of the screening program. Throughout the program, the mammograms are viewed on a SecurView mammography screening workstation (Hologic Inc.) with dual-head 5-megapixel monitors (Coronis 5MP Mammo, MFGD 5621 HD, Barco). The mammography monitors used are also tested by the physicists of the LRCB, as per established protocols.

Although the Dutch National Breast Cancer Screening Program involves independent double reading of all cases with consensus or arbitration for disparate decisions, ROCS is focused on individual reader performance. This is because the overall aim of this endeavor is to gather the evidence needed to inform the setting of new performance guidelines for radiologists. Therefore, all performance estimates are performed at the level of individual readers, and then these are aggregated into results for the screening program as a whole. In addition, due to the nature of screening, all acquisition and analysis are performed at the per case level, as opposed to per breast or per lesion.

For ROCS, a total of 40,000 cases were needed and were aimed to be included, as determined by the sample size calculation, described below. All cases included for interpretation were eligible for inclusion, with no exclusion criteria. There was no attempt to order the cases or to balance the case sets for different properties of the screened women.

### Study radiologists

All radiologists participating in the Dutch National Breast Cancer Screening Program were considered eligible, since they already have to meet minimum training, continuing certification, and case volume requirements (*n* > 3,000 screens per year) to participate in the program. To maximize the diversity in geographic, socio-economic, and other factors in the screening cases, radiologists from five different reading centers around the country were targeted to participate in ROCS. In total 21 radiologists were recruited to participate in the study, since we aimed to recruit at least 20 radiologists.

### Data acquisition

During interpretation of each case of a screening batch, a case-based PoM rating was obtained from the interpreting radiologist after their review of the case with minimal disruption of the workflow. This had to be achieved without modifying the image review software, due to certification requirements for medical software. In addition, it was not feasible to have a research assistant recording, manually or electronically, case numbers and PoM ratings from the readers during their image interpretations.

**Fig. 1:**
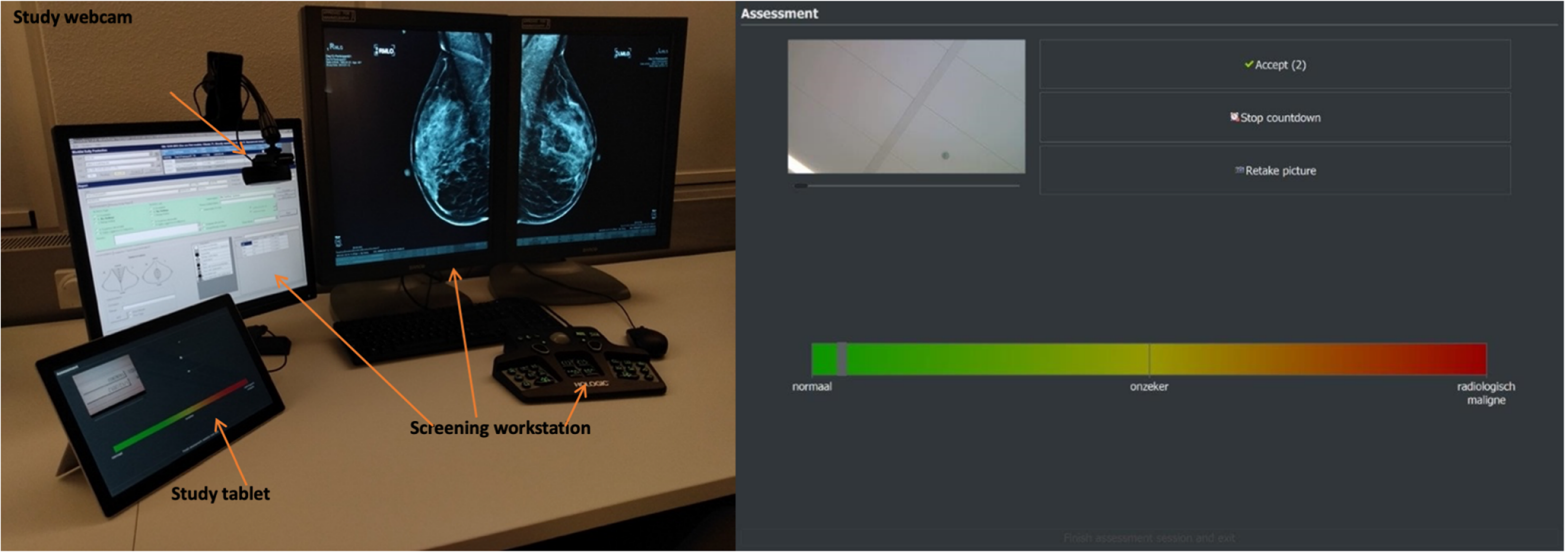
(Left) ROCS trial hardware setup, showing the tablet the radiologists use to input their determined PoM rating and the webcam aimed at the third monitor to record the case identification number. (Right) The tablet graphical user interface used to record the PoM on the colored horizontal bar, also showing the image to be captured by the webcam on the top left corner for confirmation that the camera is correctly positioned and in focus. Note that the mammogram shown in this figure has been de-identified

Therefore, a simplified graphical user interface (GUI) on a tablet (Surface Pro 4, Microsoft Corp.) was developed and implemented for this study (Fig. 1). The interface consists mainly of a horizontal bar across the entire touchscreen-enabled tablet, which the reader swipes vertically across at the horizontal position that reflects their level of suspicion of a malignancy being present. If the case raises no suspicion, then the reader will swipe vertically across the left-most region of the bar, labelled “normal,” while certainty of cancer present would result in a vertical swipe across the right-most region, labelled “malignant.” The center of the bar is labelled “uncertain.” The horizontal location of the swipe is encoded as a quasi-continuous score ranging from −100 (*normal*) to +100 (*malignant*), with zero in the center (*uncertain*), resulting in 201 possible PoM scores. This large rating scale was patterned after prior work [9]. The radiologists were instructed to swipe to the left of center if they did not recall the case, and to the right of center if they did. By displaying only the bar with the three descriptors with no numbering, and the bar consisting of a continuous gradient from green to red color, to the reader the scoring scale consisted of a continuous scale. The encoded scores are then automatically saved by the tablet.

To ensure that the recorded ratings are paired with the correct screening case, the tablet is connected to a webcam (C922 Pro Stream Webcam, Logitech), positioned so that a photograph of the area of the screen that displays the *Invitation Number* is acquired. To minimize the added process, no confirmation of the PoM selection nor tapping on a *Save* nor *Next* button is necessary. Once a rating is entered, the GUI counts down three seconds, and unless the user stops the countdown, the data is saved, and the GUI is ready for the next case. If the radiologist wanted to change the rating entered, they were instructed to re-enter a rating, and only the last one given for the same case would be considered. Together with the photo and the PoM rating, the program automatically records the time the rating is entered, allowing for analysis of timing.

A pilot test was performed by having one participating screening radiologist use the tablet during one of his regularly scheduled screening reading sessions. The session involved the reading of 164 cases, and aside from the use of the tablet as described, all other characteristics of the session were normal. Comparison of the total time for this session to previous ones resulted in an estimated slow-down by the use of the tablet of 8%.

### Study endpoints

The primary endpoint of ROCS is the estimation of the curve describing the relationship between recall rate and cancer detection rate for the average reader in the Dutch National Breast Cancer Screening Program. From this curve, it would be possible to determine the benefit, or lack thereof, in increased number of cancers detected at screening due to an increase in the recall rate. In addition to the PoM ratings from the exam interpretations, constructing this curve involves obtaining the outcome of the cases recommended for recall by each participating radiologist. This data is available through the screening organization. As per the programmatic definition, breast cancer diagnosed within 12 months of the woman being recalled from screening is defined as a screening-detected cancer.

As related secondary endpoints of the ROCS trial, two different average-reader ROC curves will be estimated to better characterize performance of readers in the screening program. The first will be constructed considering all cancers as defined by the program, i.e., all breast cancers diagnosed within 24 months after the date of screening acquisition. Interval cancer cases will be identified through linkage with the Netherlands Cancer Registry after the necessary waiting period. However, it is known that approximately half of all interval cancers are visible at the prior screening [10]. To identify these, all interval cancer cases will be reviewed by a panel of experts and graded as the cancers being *not visible*, *present with minimal signs*, or as *missed*. This is a common procedure in the Dutch National Breast Cancer Screening Program, performed for education and quality assurance purposes. Additional ROC curves will be constructed, one excluding interval cancers deemed *not visible* and one involving only invasive cancers.

### Description of the model for determining sample size

A model of breast-cancer screening was derived and used to evaluate the number of radiologists and screening exams that would generate a RR-CDR difference curve of sufficient precision. The model is only briefly described here, but see the accompanying online supplement for a thorough description of the parametric form of the model, the data used to fit the model parameters, and the model manipulation used to generate a RR-CDR difference curve. Conceptually, this curve is generated by changing the PoM score used as the threshold for recall and comparing the RR and CDR to the nominal values using a PoM threshold of 0, which represents screening as it is currently practiced. Changing the PoM threshold allows us to predict the effect of a change in RR on CDR, and to predict the precision of the difference estimates for specified numbers of readers and cases. The values for the model parameters were estimated using data from the Dutch National Breast Cancer Screening Program during the period from 2010 to 2015.

**Fig. 2:**
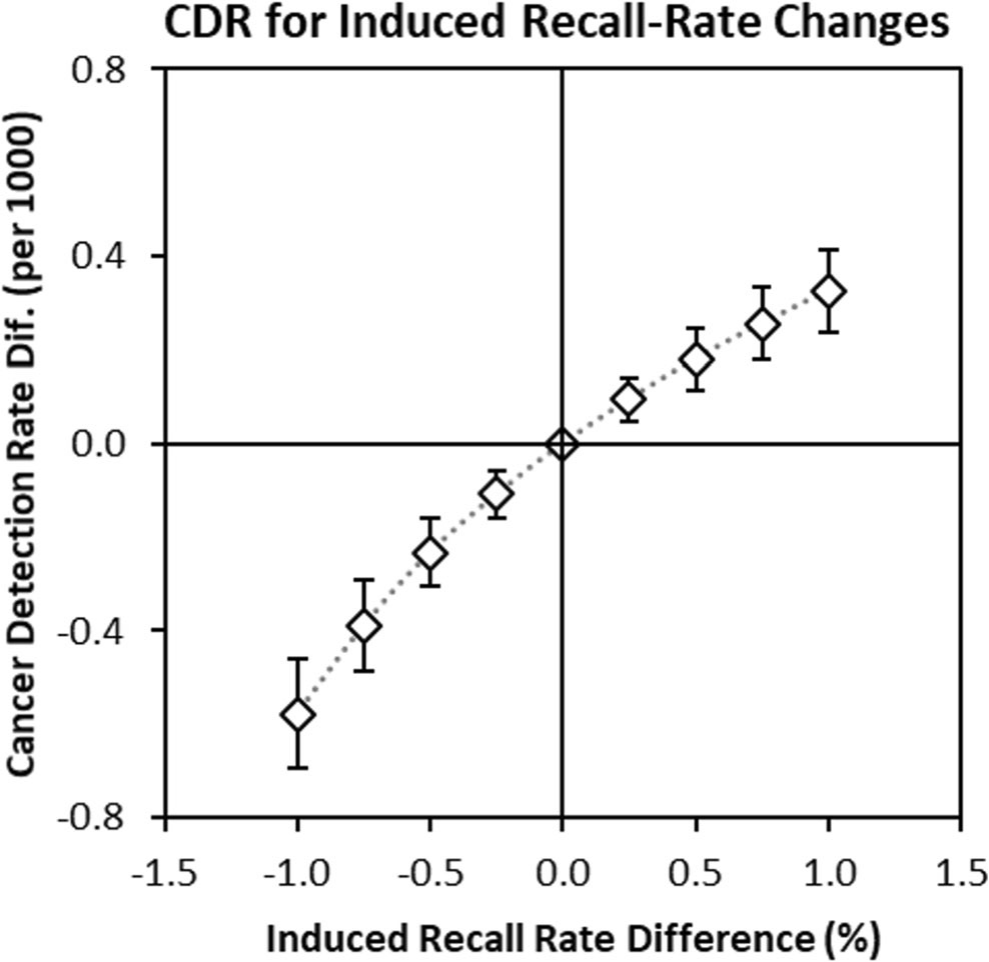
Model-based estimates of cancer-detection rate changes as a function of recall-rate changes induced by changing the PoM threshold used for recall. The error bars show the expected 95% confidence interval from precision estimates across the 20 simulated readers (see online supplement).

Using this model, Fig. 2 shows the CDR prediction and the resulting uncertainty of the estimates based on acquiring data from 20 radiologists, each reading 2,000 screening exams. As described above, this represented a large but feasible study that could be completed in a reasonable time period, and so we assessed the adequacy of this sample size. The figure assumes that the recall rate is “induced” by adjusting the PoM threshold for recall to achieve a fixed reduction in recall rate (e.g., reduce recall by 0.5%). The resulting curve is given in terms of differences with the nominal rates (RR = 2.37%, CDR = 5.53/1000) with error bars representing the expected 95% confidence interval of these differences. For changes in the recall rate of up to 1%, the standard deviation of the CDR difference is 0.12/1000 or less. This level of precision in the difference justified commencing the study.

### Data analysis

The PoM ratings for each radiologist will be used to construct recall and detection rate curves similar to those shown in Fig. 2, as well as traditional ROC curves, using well-established software for this task [11–15]. The radiologist-specific curves can then be averaged into a curve representing estimated group performance across different thresholds for recall. In addition to the visual presentation of the curves, utility analysis can be used as a rigorous approach to determining the optimal operating point on the ROC or recall/detection rate curves [16–19].

## Results

To date, the data acquisition at the reading centers has been completed. In total, 21 screening radiologists from five screening centers participated in the study between 19 March 2019 and 6 June 2019, resulting in the acquisition of a total of 42,215 recorded radiologist PoM scores and timestamps. The average number of records per radiologist was 2,010 entries (range: 954–2,842), with 14 radiologists recording more than 2,000 cases. The completion date for each radiologist varied, depending on their reading volume during the study period. In addition, the final total number of cases scored by each radiologist varied, since whole sessions were recorded, instead of data accrual stopping mid-session if 2,000 cases were reached.

**Fig. 3:**
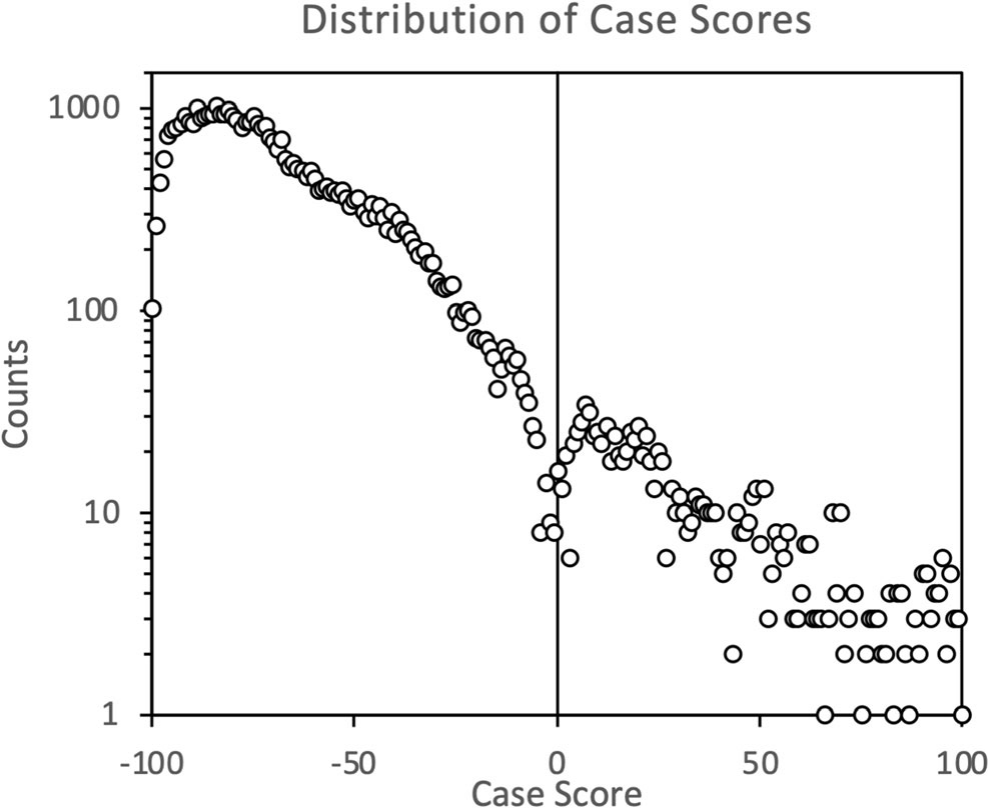
Distribution of the case scores for all 42,215 records obtained during the ROCS trial. Note that the *y* axis is plotted in logarithmic scale. The plot shows that an adequate distribution of scores was obtained across the entire available scoring range, and hence the potential problem of having all scores being clustered at the two ends, at −100 and +100, was avoided

The distribution of the recorded scores is shown in Fig. 3. An adequate distribution across the entire available scoring range was obtained, which is crucial to be able to construct adequate recall/detection rate and ROC curves. It should be noted that the number and distribution of scores shown here are based on the entirety of the records obtained. Therefore, it is expected that a small percentage of all the obtained records will have to be excluded from the final analysis due to a number of different issues, such as inability to obtain the final outcome for cases, among other issues.

The final results of the trial will be available once the short- and long-term waiting periods for collection of the actual outcomes are known.

## Discussion

The ROCS data acquisition approach acquires PoM scores and timestamps from radiologists in actual practice as they read screening mammograms. The use of external hardware, in this case a tablet and a webcam, allows for recording this data without having to modify protected and often proprietary workstation software to record a rating and timestamp per case. A simple GUI that only involves swiping on a touchscreen to enter the response, with automatic assignment of a timestamp, results in minimal intrusion of the workflow. A photographic confirmation of the case identifier avoids the need for the reader to confirm the entry or to manually move to the next case, further minimizing the disruption of the workflow. The participating radiologists reported that the procedure was not intrusive to their work.

The ROCS data collection procedure is well suited for high-volume image interpretation studies. In national screening programs, as in the Netherlands, this makes feasible the collection of such data from multiple sites for comparison. The flexibility of locating and aiming the webcam at any area of a computer monitor should allow for a similar setup even in more heterogeneous reading environments. Given that the rest of the data collection procedure is independent of the review workstation setup, the approach used in the Netherlands should be feasible for similar studies based on case ratings acquired during practice. As a result of this study, it will be possible to evaluate the tradeoff between recall rate and cancer detection rate, based on actual screening data, avoiding, or at least minimizing, any “laboratory” effect [7].

One alternative to this approach would be to construct a curve from a plot of RR/CDR pairs collected from an international group of screening programs. Since different programs operate with differing recall rates, this would give some indication of the tradeoff between recall and detection rates. However, it has been shown that there is no association between recall and detection rate internationally [20], although such association can exist within one program [21]. A variety of factors, such as screening interval, cultural differences, diagnostic infrastructure, and the medico-legal environment, impact recall and cancer-detection rates for a program, region, or country. This makes estimation of a RR/CDR curve from international data problematic.

Once such an RR-CDR and/or ROC curve is constructed, then the optimal operating point for the program can be selected based on outcomes in a data-driven manner. Knowledge of the optimal operating point could then be used to develop guidance for radiologists regarding their recall rates. This process assumes that radiologists are willing to implement these recommendations. But the change in recall rates after the recommendations derived from the Otten et al study supports the idea that Dutch radiologists are capable of adjusting their performance in response to screening program priorities. In addition, the Netherlands is evaluating the possibility of a transition to digital breast tomosynthesis and/or incorporation of artificial intelligence–based approaches for breast-cancer screening, as in other countries throughout the world. Therefore, in the future there will likely be a need to re-evaluate the trade-off between cancer detection and recall rate for new screening methodologies. If so, it would be ideal to undertake that evaluation having a clear understanding of this same trade-off in the current implementation of breast-cancer screening.

## Supplementary Information

ESM 1 (DOCX 190 kb)

## Abbreviations

CC: Cranio-caudal
CDR: Cancer detection rate
CI: Confidence interval
DM: Digital mammography
GUI: Graphical user interface
MLO: Medio-lateral oblique
PoM: Probability of malignancy
ROC: Receiver operating characteristic
ROCS: *Recall and detection Of breast Cancer in Screening*
RR: Recall rate

## Declarations

### Guarantor

The scientific guarantor of this publication is Ioannis Sechopoulos.

### Statistics and biometry

One of the authors, Craig Abbey, has significant statistical expertise.

### Methodology

• prospective

• experimental

• multicenter study
