# Supplementary material for: Evaluation of reader performance during interpretation of breast cancer screening: the Recall and detection Of breast Cancer in Screening (ROCS) trial study design
Source: Eur Radiol. 2022 Apr 28;32(11):7463–9. doi: 10.1007/s00330-022-08820-5 (PMC9668759; doi:10.1007/s00330-022-08820-5)
Supplement: Supplementary file 1 — (DOCX 190 kb) [file 330_2022_8820_MOESM1_ESM.docx]

**Supplementary Information: The design of the ROCS Trial**

**A. Overview**

To investigate the number of radiologists and screening exams to be included in the ROCS trial, a model of breast cancer screening interpretation and its performance was developed. The model was based on the Roe and Metz (RM) simulation model [1], and this description assumes some familiarity with the RM approach. Parameters of the model were fit to existing audit data from breast-cancer screening in the Netherlands. The model was then used to evaluate the precision of change estimates in the cancer-detection rate as a result of an induced change in the recall rate.

**B. Data**

The values for the model parameters were estimated using audit data from the Dutch National Screening Program during the period from 2010 to 2015. A total of 2.7 million reading results are included from 57 radiologists, with 295,685 results from initial screens and 2,434,544 results from subsequent screens. The radiologists were encoded using a label (AA, AB, etc.) for purposes of deidentification. Annual reading statistics for each radiologist were given, which included the total number of cases read, the number of recall recommendations, and the number of recalled cases that were subsequently found to be positive. These data were given for both initial and subsequent screens.

From this data, records from 51 radiologists who had read a minimum of 10,000 cases were used, reflecting 99.1% of all initial screens and 98.7% of all subsequent screens in the data. The large number of reads per radiologist was important for accurately characterizing the detection rate, which averaged 6.6/1000 in the initial screens and 5.5/1000 in the subsequent screens. The average recall rates were 5.86% for initial screens and 1.99% for subsequent screens. Table 1, presented below with the model fitting results, gives a summary of the data.

**C. Simulation model**

The RM model generates decision variables using a sum of random and fixed effects that model the influence of the imaging modality, readers, cases, and various interactions. We will not include modality effects here since we are only concerned with a single imaging modality (DM). We also incorporate a modification described previously [2, 3] that inflates the components of variance for positive cases relative to those for negative cases. This makes the model more consistent with observed data [4]. We use separate fits of the model to simulate decision variables for initial and subsequent screens.

The basic concept of the simulation is to generate a decision variable that represents the decision of a single reader after reading a single case, for initial and subsequent screens separately. This is directly analogous to the PoM score being recorded in the ROCS trial. If the decision variable is greater than a predetermined threshold, the reader recalls the case, and otherwise the reader returns the case to the screening pool. Let $X_{j,n}^{+}$ represent the decision variable for positive cases, with indices that represent the reader ($j$) and case ($n$), and let $X_{j,m}^{-}$ represent the decision variable for negative cases ($m$). For both decision variables, the reader index runs from $j=1,\ldots,N_{R}$. The case index runs from $n=1,\ldots,N_{R}^{+}$ for positive cases (i.e., $N_{j}^{+}$ is the number of positive cases seen by that radiologist) and from $m=1,\ldots,N_{R}^{-}$ for negative cases. The generating equations for each decision variable are then:

$$\begin{matrix} X_{j,m}^{-}=R_{j}^{-}+C_{m}^{-}+\left(RC\right)_{j,m}^{-}+E_{j,m}^{-} \\ X_{j,n}^{+}=\mu^{+}+R_{j}^{+}+C_{n}^{+}+\left(RC\right)_{j,n}^{+}+E_{j,n}^{+} \end{matrix}$$

where the $R$ variables represent reader effects, the $C$ variables represent case effects, the $(RC)$ variables represent reader-by-case interactions, and the $E$ variables represent residual variability. The constant, $\mu^{+}$, represents the effect of cancer presence on the decision variable. A larger value of $\mu^{+}$ leads to increased separation between the cancer and non-cancer decision-variable distributions.

Each of the variables on the right side of Equation , with the exception of $\mu^{+}$, is drawn as an independent normal random variable, with variance described by the RM model, with a modification to implement unequal variances between cancer and non-cancer decision variables. The variances of the decision variables are given by:

$$\begin{matrix} \text{Var}\left(X_{j,m}^{-}\right)=\sigma_{R}^{2}+w_{\text{Neg}}^{2}\left(\sigma_{C}^{2}+\sigma_{RC}^{2}+\sigma_{E}^{2}\right) \\ \text{Var}\left(X_{j,n}^{+}\right)=\sigma_{R}^{2}+w_{\text{Pos}}^{2}\left(\sigma_{C}^{2}+\sigma_{RC}^{2}+\sigma_{E}^{2}\right) \end{matrix}$$

These variance components are determined from the RM model, collapsing across modality-related effects, which are not present in this application. Therefore, in this case the reader variance, $\sigma_{R}^{2}$, is equivalent to the sum of the reader variance and the treatment-by-reader interaction variance in the RM model. Likewise, the case variance, $\sigma_{C}^{2}$, is the sum of the case variance and the treatment-by-case interaction variance. This condensation of the RM model preserves the desirable property that case related variances sum to 1, so that $\sigma_{C}^{2}+\sigma_{RC}^{2}+\sigma_{E}^{2}=1$. Furthermore, the weights that scale the variance for positive and negative cases are constrained so that $w_{\text{Pos}}^{2}+w_{\text{Neg}}^{2}=2$. We describe below how these weights are determined from the radiologist data.

For simulation runs of the model, we sample decision variables according to Equation , using components of variance in Equation , and compare them to a threshold, $T$. For example, if $X_{j,n}^{+}>T$, then this positive case is considered recalled, for a true-positive event. If $X_{j,m}^{-}>T$, then a false positive event is observed in the simulation. The number of positive cases seen by a given reader is simulated by sampling a binomial random variable that accounts for the total specified number of cases seen by that reader and the posited disease prevalence. Observed recall rates are simulated by computing the fraction of events exceeding the threshold for both positive and negative cases, and observed detection rates are computed as the fraction of positive events exceeding the threshold.

The parameters in the model to be fit using the audit data are the mean separation of the positive and negative distributions, $\mu^{+}$, the threshold for recall, $T$, and the ratio of the case-related variance components between the positive and negative cases, $V=w_{\text{Pos}}^{2}/w_{\text{Neg}}^{2}$. Separate parameters are estimated for initial and subsequent screens. Note that once the ratio $V$ has been specified, the scaling constants are determined by

$w_{\text{Neg}}=\sqrt{\frac{2}{1+V}} \text{and} w_{\text{Pos}}=\sqrt{\frac{2V}{1+V}}$.

The components of variance ($\sigma_{R}^{2}$, $\sigma_{C}^{2}$, $\sigma_{RC}^{2}$, and $\sigma_{E}^{2}$) are determined by the RM model. The RM model posits four different sets of variance components depending on the level of reader variance and data correlation (within-reader and between-reader correlations). We fit parameters to each of these four components of variance models, and then choose the model with the lowest residual error.

**D. Fitting the modified Roe-and-Metz model**

For each of the four RM components of variance, we fit the $\mu^{+}$, $T$, and $V$ parameters. (Note that in two of the RM variance component structures, the reader variance is dependent on $\mu^{+}$. In these cases, we have used the lowest value of the reader variance and treatment-by-interaction variance.) The parameters are fit to the screening data for Dutch radiologists described above. Table S1 gives the data used for this fitting in both initial and subsequent screening along with the resulting values from the best-fitting model in each case. The models are generally quite close to the observed data, with the largest difference coming in the standard deviation of the detection rate, where model values are lower than the data by roughly 20%. We attribute this to threshold jitter, variability in $T$, that is not accounted for in the model.

| **Table S1. Radiologist and simulation statistics**. Simulation values are shown beside the radiologist data to facilitate comparison.    **Table S2. Fitted parameters**. Best fit parameters are shown for the initial and subsequent screening data. DC: data correlation; RV: reader variability. |
| --- |

Table S2 shows the fitted parameter values. Different RM variance models lead to the best fit for the initial and subsequent screening data. The initial screening data was best fit using the high-reader-variability (RV) and low-data-correlation (DC) components, while the subsequent screening data was best fit by the high-RV and high-DC components. This may reflect the influence of prior images in the subsequent screening data, which increases the impact of the images relative to other variance components. The fitted $\mu^{+}$ is larger for the subsequent screening data than the initial data. This would also appear to reflect the availability of the prior images during the subsequent screening, which leads to increased separability in the decision variables for diseased and non-diseased cases. The fitted threshold is higher for the subsequent screening data, which is not surprising given the lower overall recall rates for these cases.

**E. Modeling sensitivity to criterion shifts**

Using the fitted model above as the basis for simulation, we can examine the effect of an induced change in recall rate on the cancer detection rate. We show this for an experiment in which 20 radiologists evaluate 2,000 cases each with different criteria. This represents the largest size study that could be feasibly conducted within the constraints of this project. The purpose of the study is to characterize the adequacy of a study of this size.

| 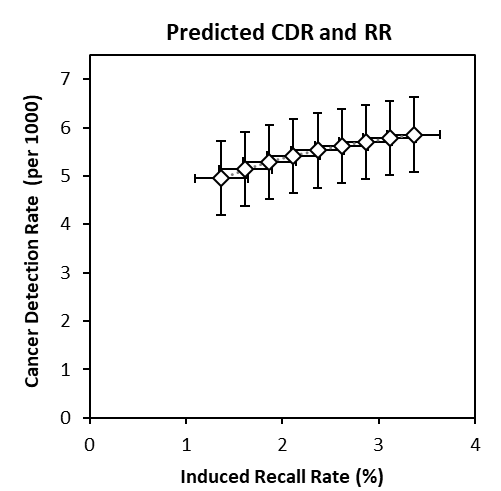  **Figure S1. Recall and Detection rate curve.** The modeled recall and detection rates for the combined initial and subsequent screens at various levels of induced recall rate changes are plotted with error bars representing the average 95% confidence interval estimated from each simulation. Note that the induced recall rate difference is a fixed quantity for these studies, but the horizontal error bars reflect the underlying sampling variability of the recall rate, which is unaffected by a fixed change. |
| --- |

Induced recall rate changes were modeled as an adjustment in the detection threshold, ($T$in Table S2) to give a fixed reduction in the recall rate. This adjustment was multiplicative, and applied to both the initial and subsequent screening models. Induced recall-rate changes of ±0.25%, ±0.5%, ±0.75%, and ±1%, across all screens (initial and subsequent) were evaluated. Figure S1 shows the CDR-RR curve at these induced recall rates along with the nominal (middle) point. The error bars, representing a 95% confidence interval on each estimate, are relatively large. However, these error bars do not account for the correlations in the estimates given the within-subjects design of the ROCS trial in which recall-rate reduction is evaluated for each reader and on the same sample of patients. When these data are converted to recall- and detection-rate changes, as in Figure 2 of the main text, there are no error bars on the x-axis, and the 95% confidence intervals on the y-axis shrink considerably. This within-subjects design is the technical motivation for the ROCS trial design.

| 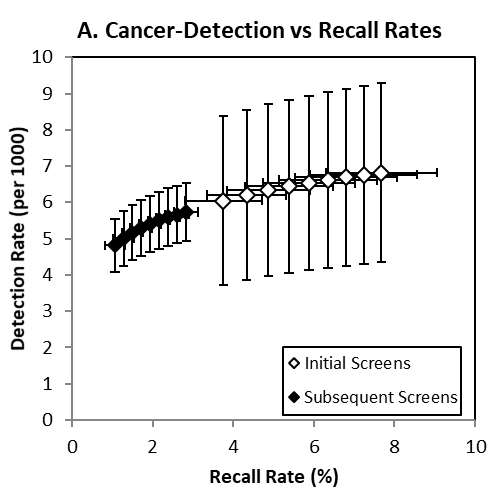 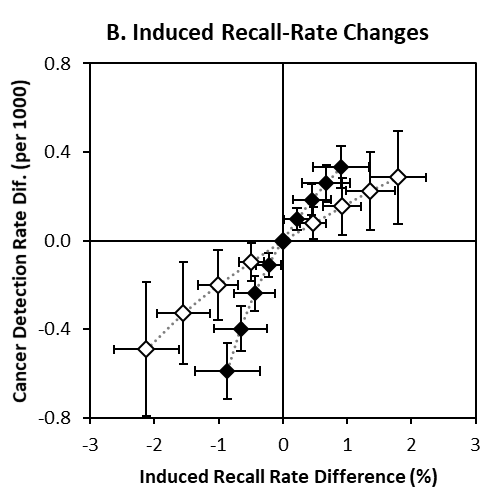  **Figure S2. CDR and RR plots stratified by case type.** (A) The CDR-RR curves show the effect of a changing threshold on initial and subsequent screens. Error bars represent a 95% confidence interval on the estimates. (B) The CDR-RR difference curves, showing the difference from the nominal rates, respond differently to the induced recall-rate changes. Additionally, since the induce recall-rate change is defined for all cases, sampling variability in the number of initial and subsequent screens lead to variability in the induced recall rate change for these subsets. This variability is characterized by horizontal error bars (not present in Fig. 2 of the main text) representing 95% confidence intervals on the recall-rate changes. Vertical error bars represent the average confidence interval estimated across readers in each simulation. The legend applies to both plots. |
| --- |

For completeness, Figure S2 provides the corresponding evaluations of the initial and subsequent screens separately. The CDR-RR curves in Figure S2(A) show substantial differences in these performance measures also seen in the audit data (cf. Table S1). Because these data are generated by adjusting a recall threshold, they have highly correlated errors that may substantially cancel when differences in the observations are used. The CDR-RR difference curves shown in Figure S2(B) show that the induced change in the overall recall rate affects the two kinds of cases somewhat differently, with a much steeper decline in the CDR difference for subsequent screens than for initial ones.

| 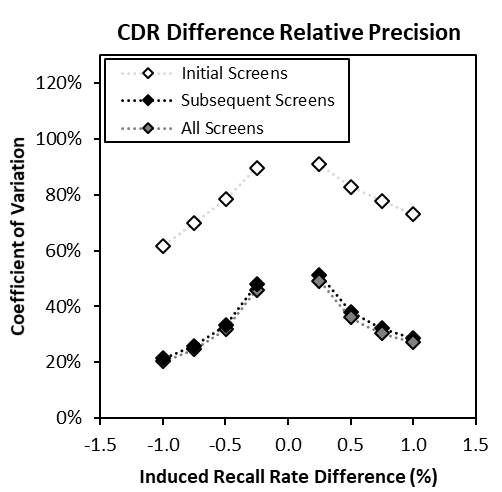  **Figure S3. Relative Precision.** The coefficient of variation (sd/mean) of the CDR difference is plotted as a function of the induced recall rate difference for initial screens, subsequent screens, and all screens combined. |
| --- |

The error bars are substantially larger for the initial screens as well, indicating the loss of precision from having fewer samples of these cases. Also, note that the recall rate differences now have error bars (in contrast to Figure 2 of the main text). This is also due to random variability in the number of initial and subsequent screens; a 1% reduction in overall recall rate will result in variable differences in the recall rates of the initial and subsequent screens if the number of initial screens varies.

To more succinctly compare the relative precision of CDR difference values, Figure S3 shows the coefficient of variation (CV), defined as the average standard deviation of the CDR difference (estimated within each simulation run by evaluating variability across readers) divided by the absolute value of the average CDR difference. This is plotted as a function of the recall rate difference to give them a common x-axis. The relatively small number of initial cases results in substantially lower relative precision in the CDR difference, which will limit the ability of the ROCS trial to characterize these. However, the number of subsequent screens are approximately a factor of two to three higher than that of initial screens, showing substantially better relative precision that will apply to the total dataset (initial and subsequent) as well. At the smallest CDR differences, where the CV is highest, it is still less than 50% for detecting a difference as small as 0.1/1000. Taken in sum, the model results suggest that the effects of criterion change can be determined with reasonable accuracy in a study involving 20 radiologists reading 2,000 cases each.

**References**

1. Roe CA, Metz CE (1997) Variance-component modeling in the analysis of receiver operating characteristic index estimates. Acad Radiol 4:587–600. https://doi.org/10.1016/S1076-6332(97)80210-3

2. Hillis SL (2012) Simulation of Unequal-Variance Binormal Multireader ROC Decision Data. Acad Radiol 19:1518–1528. https://doi.org/10.1016/j.acra.2012.09.011

3. Abbey CK, Samuelson FW, Gallas BD (2013) Statistical Power Considerations for a Utility Endpoint in Observer Performance Studies. Acad Radiol 20:798–806. https://doi.org/10.1016/j.acra.2013.02.008

4. Hillis SL, Berbaum KS (2011) Using the Mean-to-Sigma Ratio as a Measure of the Improperness of Binormal ROC Curves. Acad Radiol 18:143–154. https://doi.org/10.1016/j.acra.2010.09.002
